# Supplementary material for: The genome sequence of the Titania’s Fritillary, Boloria titania (Esper, 1793) (Lepidoptera: Nymphalidae)
Source: Wellcome Open Res. 2025 Jul 30;10:388. [Version 1] doi: 10.12688/wellcomeopenres.24643.1 (PMC12553976; doi:10.12688/wellcomeopenres.24643.1)
Supplement: Peer review history

# Reviewer report: Reviewer response for version 1

**DOI:** 10.21956/wellcomeopenres.27157.r135193

**Published:** 2026-02-28

**Authors:** Annabel Whibley

**Affiliations:** 1 Bragato Research Institute, Lincoln, New Zealand

Kleckova and colleagues report the genome assembly of Titania’s Fritillary ( *Boloria titania*). Two haplotypes are reported, with haplotype 1 scaffolded into chromosomal pseudomolecules.  Sequenced under the auspices of Project Psyche, the report follows standard genome note templating and presents the background natural history and taxonomy of the species, extensive sample metadata, comprehensive methods, clearly described and publicly available genomic resources and extensive evaluation of the quality of these resources. The reported assembly metrics are excellent in all respects (completeness, contiguity, accuracy, contamination scans, chromosome painting), formally reaching 6.C.61 for the scaffolded haplotype 1.  

 I note that specimen identification was not confirmed by barcoding and that a genome annotation has not been released at this time.

Are sufficient details of methods and materials provided to allow replication by others?

Yes

Is the rationale for creating the dataset(s) clearly described?

Yes

Are the datasets clearly presented in a useable and accessible format?

Yes

Are the protocols appropriate and is the work technically sound?

Yes

Reviewer Expertise:

Bioinformatics, Genomics

I confirm that I have read this submission and believe that I have an appropriate level of expertise to confirm that it is of an acceptable scientific standard.

# Reviewer report: Reviewer response for version 1

**DOI:** 10.21956/wellcomeopenres.27157.r135190

**Published:** 2025-10-25

**Authors:** Arun Arumugaperumal

**Affiliations:** 1 Department of Biotechnology, Rajalakshmi Engineering College, Thandalam 602105, Chennai, Tamilnadu, India

The genome note describes the whole genome sequence of *Boloria titania*, also known as the Titania’s Fritillary.  The authors have used standard Darwin's tree of life protocols to sequence the genome. The assembly reported here is of size 496.64 Mb spread among 32 chromosomes. A female specimen has been used and the Z and W sex chromosomes have been captured. The mitochondrial genome of size 15.17 kb has also been sequenced. But the annotation results are not available as Ensemble release has not happened yet. The assembly has confirmed its quality aligned with the earth biogenome project quality standards. BUSCO completeness of 98.7% indiccates that the genome is almost complete with reference to the sequences available in the databases. 

 The EBP summary metric has been mentioned as 6.C.Q61 in text. But in Table 4 it has been mentioned as 6.7.Q61. Kindly correct this mistake. Then the article can be indexed.

Are sufficient details of methods and materials provided to allow replication by others?

Yes

Is the rationale for creating the dataset(s) clearly described?

Yes

Are the datasets clearly presented in a useable and accessible format?

Yes

Are the protocols appropriate and is the work technically sound?

Yes

Reviewer Expertise:

Bioinformatics; Genomics

I confirm that I have read this submission and believe that I have an appropriate level of expertise to confirm that it is of an acceptable scientific standard.

# Reviewer report: Reviewer response for version 1

**DOI:** 10.21956/wellcomeopenres.27157.r135187

**Published:** 2025-10-25

**Authors:** Aqeel Alyousuf

**Affiliations:** 1 University of Basrah, Basrah, Iraq

The **Methods** section is technically comprehensive and well-documented, covering all major stages of the workflow, including sample acquisition, DNA extraction, library preparation, sequencing, assembly, curation, and quality assessment. The application of the Wellcome Sanger Institute (WSI) protocols, together with PacBio HiFi and Arima Hi-C technologies, reflects a high technical standard. The inclusion of detailed information on fragment size, concentration, and purity is also commendable.

**Minor Issues:**

- In the **Species taxonomy** paragraph, *Clossiana* is generally treated as a subgenus or synonym within *Boloria* in modern classifications. Listing “ *Boloria; Clossiana; Boloria titania*” without clarification is ambiguous and could be interpreted as a taxonomic error. Please revise to a consistent format, such as *Boloria (Clossiana) *or *Boloria *(syn. *Clossiana*). 
- Please correct the scientific name ( *Boloria titania*) in **Figure 1** and in the **Keywords** section.
- The **reference **“Wagner W: *Boloria titania*. 2005.” is incomplete and does not conform to standard citation guidelines. It should include the full source details, such as database name, URL, and access date.

Are sufficient details of methods and materials provided to allow replication by others?

Yes

Is the rationale for creating the dataset(s) clearly described?

Yes

Are the datasets clearly presented in a useable and accessible format?

Yes

Are the protocols appropriate and is the work technically sound?

Yes

Reviewer Expertise:

Entomology

I confirm that I have read this submission and believe that I have an appropriate level of expertise to confirm that it is of an acceptable scientific standard.
